# *Nostoc sphaeroides* Kütz Polysaccharide Improved Constipation and Promoted Intestinal Motility in Rats

**DOI:** 10.1155/2021/5596531

**Published:** 2021-07-27

**Authors:** Yinlu Liu, Litao Yang, Cuicui Bi, Kun Tang, Bo Zhang

**Affiliations:** ^1^Beijing Key Laboratory of Bioactive Substances and Functional Foods, Beijing Union University, Beijing, China; ^2^Langfang Teachers College, Langfang, China

## Abstract

Natural products and medicinal foods have attracted more and more attention because of their potential prevention and inhibition effect on constipation. *Nostoc sphaeroides* Kütz Polysaccharide (NSKP) polysaccharide is a natural product rich in polysaccharides. This work attempted to prove the effects of aqueous extracts of NSKP on STC treatment and to determine the possible mechanisms by a loperamide-induced slow transit constipation (STC) model. The results show that, in rats of the NSKP group, compared with the model group, the colon propulsion rate was improved, the time of the first grain of black stool was shortened, and the fecal wet weight was increased remarkably. The 5-HT levels were increased, but the VIP and NO levels were reduced dramatically. The number of interstitial cells of cajal (ICC) was increased by c-kit/SCF signal pathway, and the intestines were moisturized; then, constipation was relieved. It is interesting to note that NSKP appeared to be effective on constipation, so further experiments are necessary to clarify the exact mechanisms involved.

## 1. Introduction

Constipation is a common chronic gastrointestinal disease, often accompanied by symptoms of dry stool, difficulty in excretion, and the defecation frequency is less than three times a week [1–3]. Constipation can be divided into organic constipation and functional constipation (FC). Organic constipation is a kind of pathological constipation caused by endocrine diseases, digestive tract diseases, and nervous system diseases. Functional constipation is mainly caused by intestinal dysfunction, with no obvious organic disease, or is secondary to metabolic disease, systemic disease, or functional defecation difficulty due to drug factors. FC includes three types: slow transit constipation (STC), outlet obstruction constipation, and mixed constipation. Slow transit constipation is the most common symptom [4, 5]. Epidemiological studies have shown that 2%–20% of people in the world have symptoms of constipation. The prevalence rate of STC in China is about 6%, the ratio of males to females is 1% and 2%, and the trend is increasing year by year [6, 7]. There are many reasons for slow transit constipation, including insufficient dietary fiber and liquid intake, poor eating habits, and lack of exercise. At present, research on bioactive substances with laxative effects is emerging in the food and pharmaceutical industries. Natural bioactive substances usually have better curative effects in disease control and prevention and are deeply liked by consumers because of their low toxicity and fewer side effects [8–11].

*Nostoc sphaeroides* Kütz (NSK), belongs to a cyanobacterium, is a kind of traditional medicinal and edible alga, which has a long edible history in China and is widely distributed in Hubei and Sichuan provinces [12]. NSK is rich in bioactive substances, including polysaccharides, proteins, amino acids, vitamins, and minerals, among which the polysaccharide content is as high as 60%. Moreover, the polysaccharide is mostly *β*-1, 3 and *β*-1, 4 glycoside bonds, which are not easy to be absorbed by the intestinal tract, and it has strong moisture absorption, moisture retention, and high relative viscosity [9, 13, 14]. Studies have shown that NSKP has rich biological activities, including anti-inflammatory and lipid-lowering effects [15–17]. However, there are a few studies on moistening intestines and the laxative effect of NSKP. The purpose of this study was to observe the effect of NSKP on the rat model of loperamide-induced constipation. Parameters such as body weight, stool weight, and water content of rats were determined. We found evidence that NSKP has an anti-constipation effect.

## 2. Materials and Methods

### 2.1. Chemicals and Reagents

NSKP was provided by Hunan Yandi Biological Company. The method for preparing the NSKP was carried out according to the following: the dried and crushed NSK powder was extracted with 500 volumes of distilled water at 100°C for 2 h, and the supernatant was collected at 9000 g for 15 minutes. After condensed 10 times, the supernatant was precipitated overnight in 40% (v/v) ethanol at 4°C, the sediment was collected by centrifugation at 9000 g for 15 minutes, and washed with 45% (v/v) ethanol four times. The sediment was dissolved in distilled water and precipitated overnight in 40% (v/v) ethanol at 4°C again. After centrifuging 9000 g for 15 minutes, the sediment was collected, washed with 45% (v/v) ethanol twice, freeze-dried, and pulverized into NSKP powder [14].

Preparation of the experimental ink: 100 g Arabic gum was added into 800 ml water, boiled into a homogeneous sol; then, 50 g activated carbon (powdered) was added, with adding water to 1000 mL into experimental ink. The experimental ink was stored at 4°C.

ELISA kits for 5-HT (ml003125-2) were purchased from Shanghai Meilian Biological Engineering Co. Ltd. (Shanghai, China). ELISA kits for VIP (ml001911-2) were purchased from Shanghai Meilian Biological Engineering Co. Ltd. (Shanghai, China). ELISA kits for NO (ml268014-2) were purchased from Shanghai Meilian Biological Engineering Co. Ltd. (Shanghai, China). Total RNA extraction regent, SYBR Green Master primer, and oligo (dT) 18 were obtained from Roche (Basel, Switzerland). Anti-bodies against c-kit and SCF were acquired from Wuhan Servicebio Co., Ltd. (Wuhan, China).

The JJ-12J dehydrator was from Wuhan Junjie Electronics Co., Ltd.; JB-P5 embedding machine, Wuhan Junjie Electronics Co., Ltd.; RM2016 pathological slicer, Shanghai Leica instrument Co., Ltd.; JB-L5 frozen platform, Wuhan Junjie Electronics Co., Ltd.; KD-P organization spreader, Zhejiang Jinhua Cody Instrument and Equipment Co., Ltd.; and NIKON ECLIPSE E100 positive optical microscope, Nikon, Japan.

### 2.2. Animals and Feeding

Fifty SPF male rats were purchased from Beijing Weitong Lihua Experimental Animal Technology Co., Ltd. (SCXK (2016-0006), NO1100111911076580) at 8 weeks of age. The rats were placed in an SPF animal room with a temperature of 22 ± 2°C, humidity of 40%～70%, light and darkness of 12 hours a day.

### 2.3. Experimental Scheme

All animal procedures comply with the regulations of the Animal Care and Use Committee of Beijing Union University. After one week of adaptive feeding, the rats were randomly divided into five groups: control group, model group, 0.2 g/kg group, 0.4 g/kg group, and 0.8 g/kg group (*n* = 10 per group, one rat per cage). The experiment lasted for five weeks, and the animals were orally administered every day (20 mL/kg BW). From the first week to the third week, the NSKP group rats were given intragastric administration of NSKP (0.2 g/kg, 0.4 g/kg, and 0.8 g/kg), and the control group and the model group rats were given the normal saline (20 ml/kg BW). The fourth and fifth weeks: the NSKP group rats were given loperamide hydrochloride (4 mg/kg) at 7 : 00 every morning and NSKP (0.2 g/kg, 0.4 g/kg, and 0.8 g/kg) 2 hours later. The model group rats were given loperamide hydrochloride (4 mg/kg) at 7 : 00 every morning and normal saline (20 ml/kg BW) 2 hours later. The control group were given the normal saline (20 ml/kg BW). After fasted for 16 hours, animals were intraperitoneal injection of barbital at 9 : 00 a.m. Blood and colon tissue samples are collected and stored at −80°C.

### 2.4. Analysis of Food Intake, Water Intake, Body Weight, and Fecal Index

The body weighed and the feed intake were weighed every week. The fecal indexes, including the fecal wet weight and dry weight, as well as the weight per grain of feces were measured. The fecal wet weight was collected and recorded as the wet weight W1; then, the wet fecal was dried at 80°C for 3 hours and recorded as the dry weight W2. The fecal water content is calculated as follows:

(1) $$\begin{array}{c} \text{fecal water content}\left(\%\right)=\frac{W_{1}-W_{2}}{W_{1}}\times 100\%. \end{array}$$

### 2.5. Intestinal Motility Test

The test was conducted in the 5^th^ week. After fasted but drank freely for 16 hours, the rats (control group and the model group) were given corresponding doses of ink (20 ml/kg BW), but the rats (NSKP groups) were given an equal dose of ink containing the corresponding NSKP (0.2 g/kg, 0.4 g/kg, and 0.8 g/kg) by gavage. The time of the first black stool is observed.

### 2.6. Hematoxylin and Eosin Staining

The rats were killed by bloodletting of the femoral artery. The distal colon tissue of rats was cleaned with normal saline and put into a 4% tissue fixation solution for fixation, dehydration, transparency, wax dipping, and embedding to make wax blocks and paraffin sections. These paraffin sections were stained with hematoxylin and eosin (H&E, Servicebio, China). Morphological features of these sections were observed under light microscopy, after which the villus length, crypt thickness, and muscle thickness were measured using Leica Application Suite (Leica Microsystems, Switzerland). The neutral gum was sealed and observed under a microscope.

### 2.7. Enzyme-Linked Immunosorbent Assay (ELISA)

Take rat serum and colon tissue, wash colon tissue with normal saline, grind to prepare 10% tissue homogenate, centrifuge the supernatant for 20 minutes, and then determine the contents of 5-HT, VIP, and NO in serum and tissue homogenate according to ELISA experimental instructions.

### 2.8. Immunohistochemical Analysis

The colonic tissue of rats was washed with PBS, fixed with 4% paraformaldehyde, routinely embedded, sliced, dewaxed, hydrated, and repaired with antigen. c-kit first antibody was added and incubated at 37°C for 2 hours and overnight at 4°C. The biotin-labeled secondary antibody and peroxidase solution were added dropwise for 15 minutes, and DAB was developed for 10 minutes. It is usually dehydrated and transparent, and the film is mounted. CKX41 inverted fluorescence microscope showed that the intestinal tissue section showed specific brown staining as positive. Analysis: randomly select each slice in each group to fill the whole field of vision of the tissue as far as possible to take pictures at 400 times the field of view and ensure that the background light of all photos is consistent. Histochemistry score (H-score): H-score is a histological scoring method to deal with immunohistochemistry, which converts the positive number and staining intensity in each section into the corresponding value, so as to achieve the purpose of semiquantitative tissue staining. H-score (H-score = ∑(*pi* × *i*) = (percentage of weak intensity area × 1) + (percentage of moderate intensity area × 2) + (percentage of strong intensity area × 3). In the formula, pi represents the area ratio of positive signal pixels and *i* represents coloring intensity). H-score is the data between 0–300, the larger the data, the stronger the comprehensive positive intensity.

### 2.9. Western Blot Analysis

The appropriate concentration of SDS-polyacrylamide gel was selected according to the size of the protein to be detected for electrophoresis (40 *μ*g/swimming lane). After electrophoresis swimming, the separation gel was obtained. First, put the PVDF membrane and the sponge in the transfer buffer, mark the PVDF film, place the cut membrane on the semidry rotation membrane instrument, and wet the film with the electric buffer to transfer the film. The PVDF membrane was transferred to the washing box containing phosphate buffer, washed 3 times (10 min/times) with an appropriate amount of TBST, incubated with secondary antibody (1 : 200) for 2 hours, washed 3 times (10 min/times) into the washing box containing phosphate buffer solution, developed, and photographed. *β*-Actin was used as an internal reference.

### 2.10. Quantitative Real-Time Polymerase Chain Reaction

Total RNA from rat colon tissues was extracted using a total RNA extraction kit (Servicebio, China) according to the manufacturer's protocol. Two micrograms of total RNA samples was used to synthesize cDNA using the Revert Aid First Strand cDNA Synthesis Kit (Thermo Scientific, USA). Quantitative real-time reverse-transcription PCR (qRT–PCR) was performed in triplicate using SYBR Green and a Light Cycler 480 Real-Time PCR System (Roche Diagnostics). Each well was loaded with a 20 *μ*L sample, containing 2.5 *μ*L cDNA, 2.0 *μ*L target primers, 8.0 *μ*L water, and 12.5 *μ*L Kapa SYBR Fast Master Mix. Hot-start PCR was performed for 40 cycles. Each cycle consisted of denaturation for 15 s at 95°C, annealing for 30 s, and elongation for 30 s at 60°C. Roche Light Cycler software (version 1.5.0, Roche Diagnostics) was utilized for data analysis. The results were analyzed using the 2^−ΔΔCt^ method of analysis. Mean expression levels for control group rats were set as 100%. The primers used are shown in Table 1.

### 2.11. Data Analysis

Results are represented as the mean ± SEM. One-way analysis of variance (ANOVA) and Newman–Keuls post hoc tests were performed to compare differences between groups by using SPSS version 22.0. *P* < 0.05 was considered statistically significant.

## 3. Result

### 3.1. Effects of NSKP on Body Weight, Feed Intake, and Food Utilization Rate

**Figure 1:**
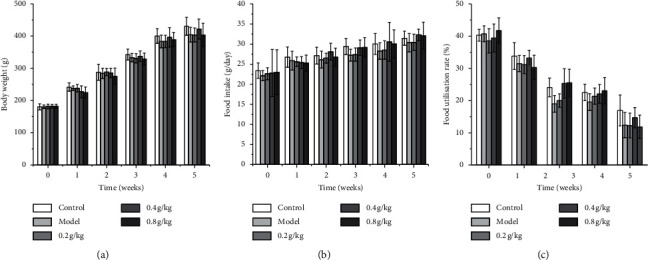
Effects of NSKP on body and feeding behavior in loperamide-induced constipated rats. (a) Body weight. (b) Food intake. (c) Food utilization rate. Bars marked with different letters represent statistically significant difference (*P* < 0.05), whereas bars labeled with the same letter indicate no statistically significant difference between the groups (*P* > 0.05). Values represent mean ± SEM; *n* = 10 in each group.

There was no significant difference in the body weight in each group during the experiment. The feed intake and food utilization rate of rats were not significantly different among the groups. The results showed that NSKP had no significant effect on growth (Figure 1).

### 3.2. Effects of NSKP on Fecal Indexes

As shown in Table 2, compared with the model group, there were significant differences in fecal dry weight, water content, and fecal granule number in the NSKP dose group (*P* < 0.05), indicating that the constipation model was successfully established. For the dry weight of feces, there is no significant difference between the model group and the 0.2 g/kg group, but the model group is significantly different from 0.4 g/kg and 0.8 g/kg (*P* < 0.05). there was no significant difference in single fecal weight among groups (*P* > 0.05) (Table 2).

### 3.3. Intestinal Motility Test

**Figure 2:**
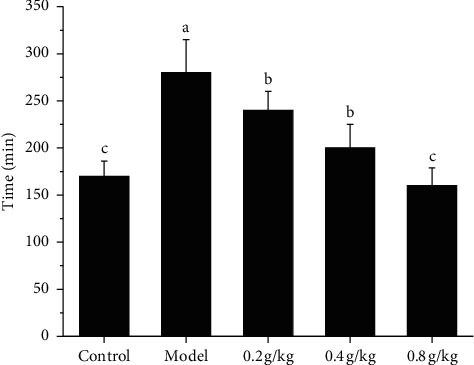
First black stool defecation time of rats after inducing constipation by loperamide. Bars marked with different letters represent statistically significant difference (*P* < 0.05), whereas bars labeled with the same letter indicate no statistically significant difference between the groups (*P* > 0.05). Values represent mean ± SEM; *n* = 10 in each group.

As shown in Figure 2, the time of producing the first black stool in the model group was significantly longer than that in the control group and NSKP dose group. However, there was no significant difference between the 0.8 g/kg group and the control group (Figure 2).

### 3.4. Effect of NSKP on Histological Structure of the Colon

**Figure 3:**
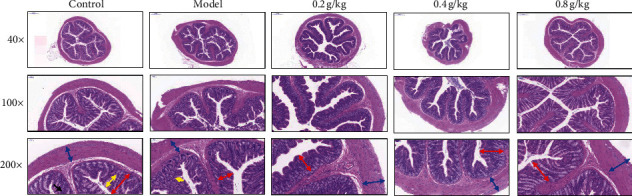
Alterations in the histological structure in lop-induced constipated rat after NSKP treatment. H&E-stained sections of rat colons from the control group, model group, 0.2 g/kg group, 0.4 g/kg group, and 0.8 g/kg group were observed at two different magnifications (40×, 100×, and 200×) using a light microscope. The blue double arrow indicates the thickness of the muscle layer. The red double arrow indicates the thickness of the mucosal layer, yellow double arrow indicates the length of intestinal glands. black arrows indicate goblet cells.

**Figure 4:**
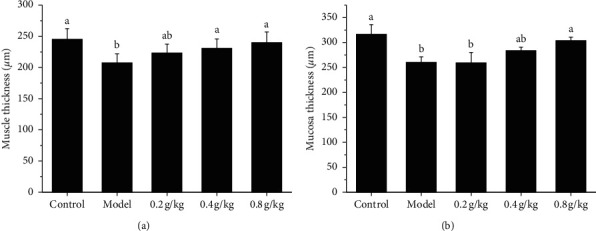
Mucosa layer thickness, muscle thickness, and crypt layer thickness are presented as graphs. (a) Muscle thickness; (b) mucosa thickness. Bars marked with different letters represent statistically significant difference (*P* < 0.05), whereas bars labeled with the same letter indicate no statistically significant difference between the groups (*P* > 0.05). Values represent mean ± SEM; *n* = 10 in each group.

The intestinal structure in each group was observed by HE staining. The colon structure of the model group is incomplete, and the mucosal layer cells are arranged disorderly, and the number is reduced, which is obviously different from the control group and 0.8 g/kg group. Compared with the thickness of the mucous layer and muscle layer in the model group, there was no significant difference in the 0.2 g/kg group, but there was a significant difference in the 0.8 g/kg group (*P* < 0.05). The length of intestinal gland epithelium length and the number of goblet cells of the model group were significantly lower than that of the NSKP group (*P* < 0.05). The number of goblet cells per unit length of the 0.2 g/kg group and the 0.8 g/kg group was significantly higher than that of the model group (*P* < 0.05), and there was no significant difference between the 0.2 g/kg group and the 0.8 g/kg group (Figures 3 and 4, Table 3).

### 3.5. The Intervention with NSKP on 5-HT, VIP, and NO

**Figure 5:**
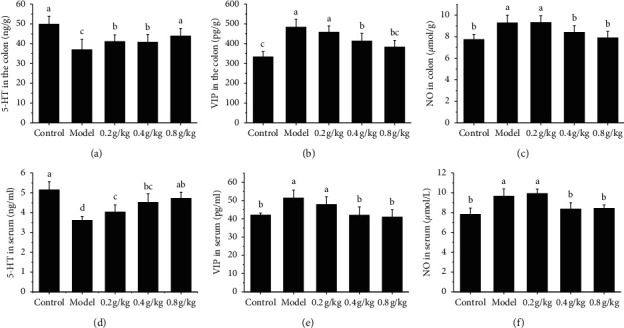
The midgut neurotransmitters 5-HT, VIP, and NO in serum and colonic tissue of rats were determined. (a) 5-HT in the colon; (b) 5-HT in colon; (c) VIP in the colon; (d) VIP in serum; (e) NO in serum; (f) NO in serum. Bars marked with different letters represent statistically significant difference (*P* < 0.05), whereas bars labeled with the same letter indicate no statistically significant difference between the groups (*P* > 0.05). Values represent mean ± SEM; *n* = 10 in each group.

5-HT, VIP, and NO in serum and colonic tissue were investigated in the colonic tissue. The results showed that the 5-HT levels of the NSKP dose group were significantly higher than those of the model group (*P* < 0.05). The content of VIP and NO in the model group was significantly higher than that in the control group, while that in the NSKP group was significantly lower than that in the model group (*P* < 0.05). There was no significant difference in the level of intestinal neurotransmitters between the 0.8 g/kg group and the control group (*P* > 0.05). The results of detection of these intestinal neurotransmitters in serum were the same as those in colon (Figure 5).

### 3.6. Effect of NSKP on Expression of ICC in Colon

**Figure 6:**
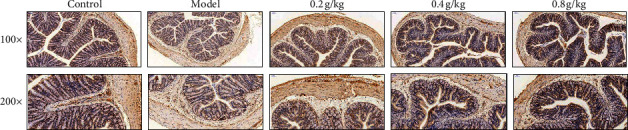
Immunohistochemical staining of the ICC by c-kit (*n* = 10, 100×, 200×).

**Figure 7:**
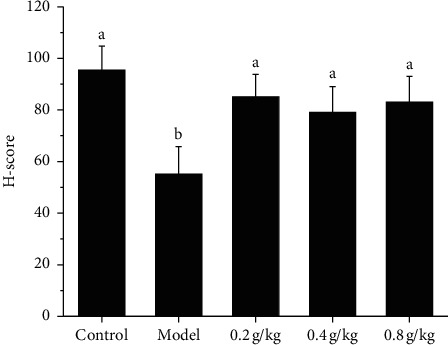
Expression of the ICC by c-kit by H-score and presented in order of increasing H-score. Bars marked with different letters represent statistically significant difference (*P* < 0.05), whereas bars labeled with the same letter indicate no statistically significant difference between the groups (*P* > 0.05). Values represent mean ± SEM; *n* = 10 in each group.

We used the immunohistochemical method to explore the expression of ICC in the colon. Under a light microscope, the ICC positive cells in the model group had smaller cell bodies, lighter staining, fewer positive granules, and lower density. The network structure of adjacent ICC positive cells was discontinuous and the distribution of ICC positive cells was the same as that of the control group, but there were some differences in staining and quantity. The ICC positive cells in the NSKP group were larger and darker than those in the control group. The distribution and staining of positive cells were similar to those in the control group. By measuring the expression of c-kit by H-score in colon, the expression of c-kit in colon of the model group was significantly different from that of NSKP dose group (*P* < 0.05) (Figures 6 and 7).

### 3.7. Effect of NSKP on the Protein Expression of c-Kit/SCF

**Figure 8:**
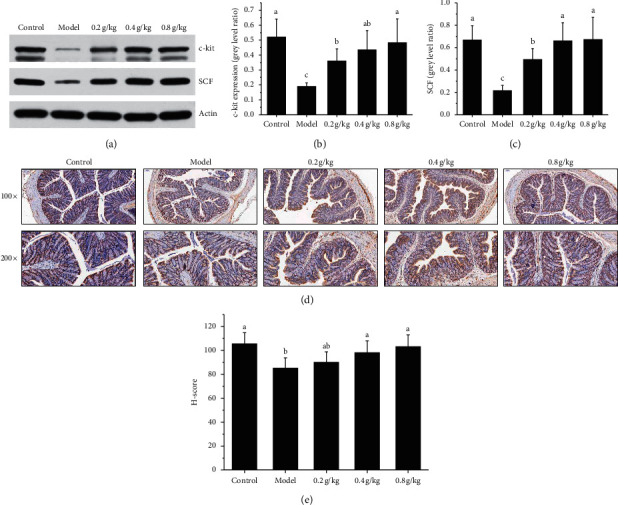
NSKP ameliorated the function of ICC. (a) SCF, c-Kit protein expression levels in the rat colon; (b) c-kit expression; (c) SCF expression; (d) P13 K expression; (e) H-score. Bars marked with different letters represent statistically significant difference (*P* < 0.05), whereas bars labeled with the same letter indicate no statistically significant difference between the groups (*P* > 0.05). Values represent mean ± SEM; *n* = 10 in each group.

We also studied the effect of NSKP on moistening intestines and relieving defecation from the expression of c-kit and SCF protein. Compared with the model group, the expression of c-kit and SCF protein in the control group and NSKP dose group was significantly weaker than that in the model group. Because the gray value error is large, the value is calculated by AlphaEaseFC software. Then, the relative expression value of each group of rats was calculated. The results showed that, compared with the model group, the expression levels of c-kit and SCF in the control group and NSKP group were significantly higher than those in the model group (*P* < 0.05) (Figure 8).

### 3.8. Effect of NSKP on mRNA Expression of c-Kit, SCF, and PI3K

**Figure 9:**
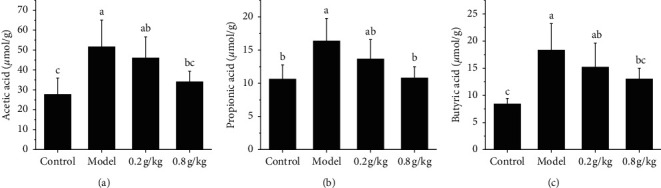
Real-time polymerase chain reaction detection of c-kit, SCF, and PI3K messenger RNA expression. (a) c-kit mRNA quantity; (b) SCF mRNA quantity; (c) PI3K mRNA quantity. Bars marked with different letters represent statistically significant (*P* < 0.05), whereas bars labeled with the same letter indicate no statistically significant difference between the groups (*P* > 0.05). Values represent mean ± SEM; *n* = 10 in each group.

In order to explore whether the treatment of NSKP can affect the regulation of muscle contraction-related mRNA, the expression levels of c-kit, SCF, and PI3K in the colon of rats with constipation were observed with specific primers. The results showed that the level of c-kit mRNA in the model group was significantly lower than that of the 0.4 g/kg and the 0.8 g/kg groups. We also detected the level expression of SCF mRNA. The expression of SCF mRNA in the model group was significantly lower than that in the control group, while the expression of SCF mRNA in the 0.4 g/kg and the 0.8 g/kg groups was increased (*P* < 0.05). In addition, we also examined the effect of NSKP on PI3K gene expression. The expression level of the PI3K gene in the control group and NSKP dose group was significantly higher than that in the model group (*P* < 0.05) (Figure 9).

## 4. Discussion

Nowadays, the prevalence rate of functional constipation is gradually increasing, and more and more people are plagued by it, especially the middle-aged and elderly people. It greatly reduces the quality of life of patients and brings a huge burden to everyone's health and economy. Natural products and medicinal foods have attracted more and more attention because they may become new drugs to prevent and inhibit constipation [18–20]. Therefore, we studied the therapeutic effect of NSKP on loperamide hydrochloride-induced constipation in rats. Loperamide, atropine-diphenoxylate, and morphine are widely used to induce constipation in laboratory animals. Among these drugs, loperamide hydrochloride is the most commonly used drug because it inhibits the secretion of water and the smooth movement of the intestinal wall, which causes long-term defecation and delays in intestinal transit [21, 22].

In this experiment, the rat model of constipation was established by intragastric administration of loperamide hydrochloride, and the effects of NSKP on the body weight, fecal weight, and water content of rats were detected. When the rat produced, the first black stool was used to observe the intestinal driving force of the rat. And by HE staining to observe the influence of indicators such as the structure of the rat's colon, the changes of serum and midgut neurotransmitters 5-HT, VIP, and NO were determined and regulating the number of ICC through c-kit/SCF signal pathway to explore the mechanism of NSKP in moistening intestines and defecation. In this study, there was no significant difference in body weight and food intake among the three groups from the beginning of feeding to the end of the experiment. After oral administration of loperamide hydrochloride, the fecal granule number, wet weight, and water content of the model group were significantly lower than those of the control group, while the fecal granule number, wet weight, and water content of different doses of NSKP groups were increased in different degrees, and even the fecal granule number, wet weight, and water content of the 0.4 g/kg and 0.8 g/kg group were better than those of the control group to some extent. In the study of intestinal propulsion of rats, the time of producing the first black stool in the model group was significantly longer than that in the control group (*P* < 0.05), while different dose groups of NSKP could restore the intestinal propulsion of rats in different degrees. Observed by HE staining, *Pueraria lobata* polysaccharides have a certain protective effect on the intestinal structure.

The enteric nervous system (ENS) is the largest peripheral nervous system of the body, which is mainly composed of the submucosal plexus and muscular plexus, in which the former mainly regulates intestinal secretion and absorption, while the latter mainly regulates intestinal peristalsis. It can be seen that ENS is the basis of intestinal physiological function, so the pathological changes of system structure and function will lead to intestinal dysfunction, resulting in the occurrence of corresponding diseases [23]. The regulation of the intestinal tract by the intestinal nervous system is through the release of a variety of different neurotransmitters by neurons, which specifically binds to receptors or postsynaptic neurons on the effector cells, and their abnormal synthesis or storage, degradation, and release abnormalities, as well as changes in the affinity and number of receptors, will lead to conduction disorders. It has been confirmed that neurotransmitters can be divided into two types: excitatory and inhibitory. Excitatory transmitters are represented by substance P (SP) and acetylcholine (ACh), while inhibitory neurotransmitters are represented by nitric oxide (NO), vasoactive intestinal peptide (VIP), and so on [24, 25]. The upregulation of the 5-HT level can accelerate intestinal peristalsis and improve the blood circulation of the intestinal wall. Clinical and animal studies have shown that the level of 5-HT is positively correlated with intestinal motility and sensory abnormalities [26, 27]. Peptidergic neurotransmitters, VIP and NO, and VIP, the main inhibitory neurotransmitters in ENS, regulate and dilate vascular smooth muscle and intestinal tract, which is beneficial to mucus secretion in the gastrointestinal tract. Studies have shown that VIP has the highest content in the gastrointestinal tract, which can reduce the frequency of colonic motility and inhibit noncholinergic transmitters and nonadrenergic neurons in the gastrointestinal tract [28]. NO is the most important inhibitory neurotransmitter, which inhibits gastrointestinal motility and is catalyzed by nitric oxide synthase (NOS) [29–31]. In this experiment, the contents of 5-HT, VIP, and NO in the serum and colon tissue of rats were determined. The results showed that the content of 5-HT in the serum and colon tissue of the model group and control group decreased significantly, while the content of VIP and NO increased significantly (*P* < 0.05). Compared with the model group, the middle and high dose group of NSKP recovered in different degrees, and the difference was significant (*P* < 0.05).

ICC are a kind of very special interstitial cells between the enteric nervous system (ENS) and smooth muscle cells. They are pacemaker cells of slow wave in the gastrointestinal tract. They play an important role in regulating the transmission of intestinal nerve signals to effector smooth muscle cells. The changes in structure, function, quantity, and distribution of ICC are the main causes of gastrointestinal motility disturbance [32, 33]. c-kit is a transmembrane protein, which is expressed in almost all ICC, so c-kit has become an important marker for detecting the existence of ICC [34, 35]. At the same time, ICC cells and stem cell factor (SCF) are closely related to c-kit/SCF signal pathway. As a signal pathway initiated by the binding of tyrosine kinase receptor family and its ligand SCF, c-kit not only plays an important role in the regulation of hematopoietic stem cells and melanocytes but also plays an important role in the differentiation and development of ICC, phenotypic maintenance, and the stability of gastrointestinal rhythmic activity [36]. SCF is a natural ligand of c-kit, expressed in various tissues of the body, but is mainly produced by bone marrow stromal cells. Each c-kit monomer binds to a SCF through extracellular domain 1-3. After SCF dimerization, the structure of c-kit monomer changes and produces homodimerization, which leads to automatic phosphorylation of amino acid residues on the cell membrane and stimulates various second signal molecules to regulate the cellular function of ICC. The second signal molecule is phosphatidylinositol 3-kinase (PI3K), an apolipoprotein that converts 4,5-diphosphoinositide into 3,4,5-triphosphoinositide through the combination with tyrosine 721 of c-kit [37]. The normal expression of c-kit and the binding of sufficient ligand SCF in the microenvironment are the key to maintain the phenotype of ICC. Any pathogenic factor that leads to the decrease of the expression of c-kit or SCF may cause the phenotypic transformation of ICC and lose its corresponding function. A large number of studies have suggested that abnormal expression of c-kit and SCF genes and blocking of corresponding signal pathways can lead to the phenotypic transformation of ICC, resulting in morphological and functional abnormalities [38, 39]. In our experiment, we used the immunohistochemical method to observe the expression of ICC in the colon and semiquantitative analysis with the H-score method. The results showed that the number of ICC in the model group was significantly less than that in the control group and NSKP group, and the difference was statistically significant. Then, we studied the c-kit/SCF signal pathway by WB and RTPCR methods. The results showed that the protein and mRNA expression of c-kit and SCF and the expression of PI3K mRNA in the model group were significantly lower than those in the model group, while that in the NSKP dose group was significantly higher than that in the model group. Therefore, SCF/c-kit may play a role through PI3K.

## 5. Conclusion

The results showed that the dose group of NSKP could shorten the first defecation time, increase the water content of feces, increase the colon propulsion rate, increase the level of 5-HT, decrease the levels of VIP and NO, and regulate the number of ICC through c-kit/SCF signal pathway to moisturize the intestines and relieve defecation. It is proved that NSKP has the potential to treat and prevent constipation. The action mechanism of NSKP may increase the content of ICC through PI3K/SCF/c-kit signal pathway, promote the production of a slow wave in the colon and regulate the rhythm of contractile activity of smooth muscle, thus, playing the role of moistening intestines and relieving defecation.

## Figures and Tables

**Table 1:** Targeted genes and internal parameter of primer.

Amplified products	Sizes		Sequence
*c-kit*	253	Forward	ATCAGGGCGACTTCAATTACGA
		Reverse	TGCTGGTGTTCAGGTTTAGGGT

*SCF*	295	Forward	TGACCTCGTGGCATGTATGG
		Reverse	GGACTTTGCGGCTTTCCTATTAC

*P13 K*	219	Forward	TGACAGGCACAACGACAACATC
		Reverse	AGGTAAGCCCTAACGCAGACAT

*β-Actin*	240	Forward	TGCTATGTTGCCCTAGACTTCG
		Reverse	GTTGGCATAGAGGTCTTTACGG

**Table 2:** Effect of NSKP on fecal indexes of rats (*n* = 10).

	Control	Model	0.2 g/kg	0.4 g/kg	0.8 g/kg
Wet weight of feces (g)	6.51 ± 1.26^ab^	5.59 ± 0.55^c^	6.21 ± 1.26^b^	7.20 ± 1.25^a^	6.79 ± 0.76^a^
Dry weight of feces (g)	4.77 ± 0.66^a^	4.33 ± 0.40^b^	4.48 ± 0.55^b^	5.09 ± 0.73^a^	4.75 ± 0.39^a^
Fecal water content (%)	26.09 ± 4.52^b^	22.43 ± 3.07^c^	26.70 ± 6.35^b^	28.83 ± 4.39^a^	29.77 ± 3.42^a^
Single fecal weight (g)	0.15 ± 0.03	0.14 ± 0.02	0.15 ± 0.02	0.15 ± 0.03	0.14 ± 0.02
Fecal grain number (ea)	44 ± 6^b^	38 ± 3c	42 ± 6^b^	50 ± 7^a^	48 ± 5^a^

Values represent mean ± SEM; *n* = 10 in each group. Superscript letters represent statistically significant differences (*P* < 0.05). Instances of the same letter between groups indicate that no statistically significant difference was found (*P* > 0.05).

**Table 3:** Crypt layer thickness and goblet cell measurement (*n* = 10).

Parameter	Crypt layer thickness (mm)	Number of goblet cells (ea)	Number of goblet cells per unit length (ea/mm)
Control	0.67 ± 0.05^a^	54 ± 7.1^a^	81.1 ± 5.3^a^
Model	0.38 ± 0.06^c^	22.2 ± 4.2^d^	58.5 ± 11.1^c^
0.2 g/kg	0.46 ± 0.07^c^	32.2 ± 3.1^c^	71.5 ± 11.3^b^
0.4 g/kg	0.52 ± 0.05^b^	36.8 ± 3.5^b^	71.8 ± 9.6^b^
0.8 g/kg	0.60 ± 0.07^a^	44.8 ± 4.8^b^	76.5 ± 11.5^ab^

Values represent mean ± SEM; *n* = 10 in each group. Superscript letters represent statistically significant differences (*P* < 0.05). Instances of the same letter between groups indicate that no statistically significant difference was found (*P* > 0.05).

## Data Availability

The data used to support the findings of this study are included within the article.
